# LIFE COURSE EXPOSURE TO RISKY WORK ENVIRONMENTS AND EARLY RETIREMENT DUE TO DISABILITY

**DOI:** 10.1093/geroni/igad104.0435

**Published:** 2023-12-21

**Authors:** Amanda Sonnega, Dawn Carr, Qize Chen, Rebekah Carpenter, Katy (Qiuchang) Cao

**Affiliations:** University of Michigan, Ann Arbor, Michigan, United States; Florida State University, Tallahassee, Florida, United States; University of Michigan, Ann Arbor, Michigan, United States; Florida State University, Tallahassee, Florida, United States; Florida State University, Tallahassee, Florida, United States

## Abstract

Jobs that are physically demanding, stressful, and dangerous are typically associated with earlier onset of physical health decline and may be associated with increased risk of early retirement due to disability. Occupations reflect differentiation by race and ethnicity due to structural racism in the educational and judicial systems, and the labor market, with Blacks and Hispanics more likely to occupy jobs that are more physically demanding and dangerous. If such jobs lead to earlier retirement, they may exacerbate wealth disparities. We use new data from the Health and Retirement Study’ Life History Mail Survey linked to rich job characteristic data from O*NET to characterize lifetime exposure to risky work environments based on levels of physiological and psychological stress. We evaluate differences by race/ethnicity in exposure to a wide range of risky work environments, explore whether they relate to disability retirement, and explore whether these associations differ by race. We show that there are large racial and ethnic differences in both the likelihood of disability retirement and lifetime exposure to risky work environments. Probit regressions controlling for education and cohort suggest that racial and ethnic differences in disability retirement are partly explained by lifetime exposure to risky work. Our findings may inform modifications to work environments that could improve the lives of workers in “bad jobs” and potentially reduce disparities in disability retirement.

